# Identification of a Novel Nonsense Mutation p.Tyr1957Ter of CACNA1A in a Chinese Family with Episodic Ataxia 2

**DOI:** 10.1371/journal.pone.0056362

**Published:** 2013-02-18

**Authors:** Yafang Hu, Haishan Jiang, Qun Wang, Zuoshan Xie, Suyue Pan

**Affiliations:** Department of Neurology, Nanfang Hospital, Southern Medical University, Guangzhou, People’s Republic of China; The Scripps Research Institute, United States of America

## Abstract

Type 2 episodic ataxia (EA2) is the most common subtype among a group of rare hereditary syndromes characterized by recurrent attacks of ataxia. More than 60 mutations and several gene rearrangements due to large deletions in CACNA1A gene have been reported so far for the cause of EA2. Because CACNA1A gene is a large gene containing 47 exons and there is no hot spot mutation, direct sequencing will be a challenge in clinical genetic testing. In this study, we used next generation sequencing technology to identify a novel nonsense mutation of CACNA1A (p.Tyr1957Ter, NP_001120693.1) resulting in truncated protein without 305 amino acids in the c-terminus. Sanger sequencing confirmed the heterozygous mutation of CACNA1A in a Chinese family with 11 affected individuals. Affected individuals experienced recurrent attacks with or without nystagmus, dysarthria, seizure, myokymia, dystonia, weakness, blurred vision, visual field defects, diplopia, migraine, dizziness, nausea and vomiting, sweating and abdominal pain. This is the first report of EA2 in a Chinese family that carries a novel mutation in CACNA1A gene and had abdominal pain as a novel phenotype associated with EA2.

## Introduction

Episodic ataxias (EAs) are rare, autosomal dominant disorders characterized by recurrent attacks of imbalance and incoordination [1]. Until now, there have been at least seven distinct subtypes of episodic ataxias based on clinical features and genetics. Mutations of four genes have been linked to different syndromes: KCNA1, CACNA1A, CACNB4, and SLCIA3 for EA1, EA2, EA5 and EA6 respectively [2], [3], [4], [5].

Type 2 episodic ataxia (EA2) is the most common episodic ataxia syndrome. EA2 typically occurs early in life and patients may variably exhibit nystagmus, vertigo, diplopia, tinnitus, dystonia, hemiplegia, dysarthria, nausea, vomiting, migraine and headache [1]. These symptoms last from minutes to days in duration. Patients with EA2 respond well to acetazolamide treatment.

EA2 is caused by heterozygous mutations in CACNA1A, which are also responsible for familial hemiplegic migraine type-1 (FHM1) and spinocerebellar ataxia type-6 (SCA 6) [3], [6]. CACNA1A gene (NM_001127221.1) is a large gene spanning approximately 300 kb with 47 exons, which encodes Cav2.1, the main subunit of the P/Q type voltage-gated calcium channel [3]. The full-length CACNA1A protein contains four domains consisting of 6- transmembrane segments, N-terminus, C-terminus, and intracellular loops [7]. Most FHM1 carried missense mutations in CACNA1A gene. More than 60 mutations including missense, nonsense, splice site, small inserts or deletions that span the entire gene have been identified for EA2 [7]. Gene rearrangement due to large genomic deletions is another important cause of EA2 and these deletions are highly variable in size and location [8], [9], [10], [11]. Glutamine-encoding CAG-repeat expansion in the C terminus of CACNA1A causes SCA6 [6].

CACNA1A is heavily expressed throughout the central nervous system and particularly enriched in axon terminals and in Purkinje cells of the cerebellum where it is involved in coupling action potentials with neurotransmitter release [12], [13], [14], [15]. It is generally thought that mutations in CACNA1A result in decreases (mostly in EA2) or increases (FMH1 or SCA6) in CaV2.1 currents [1]. The decrease in Ca2+ entry through CaV2.1 channels results in decreased output from Purkinje cells contributes to EA2 symptoms [16]. On the other hand, increased Ca2+ entry causes aberrant transmitter release and excitotoxicity.

Although EA2 is the best characterized syndrome, no CACNA1A mutation has been found in many patients who present EA2-like symptoms. Because there are no hot mutation sites for large gene like CACNA1A and overlapping clinical features among different types of ataxia, we explored next generation sequencing technology to identify mutations in all exons of four known EAs genes, KCNA1, CACNA1A, CACNB4, and SLCIA3. We have successfully identified a novel mutation of CACNA1A associated with EA2 in a Chinese family with 11 affected individuals.

## Subjects and Methods

### Subjects

A 19 year old man from the southern China area (Chinese Han) was hospitalized because of recurrent abdominal pain attacks associated with dysarthria, limb weakness, and imbalance gait with duration of 1 hour. Ten more members in his four-generation family had similar symptoms. Genetic tests were performed for the affected individuals and an unaffected individual as a control.

This study was approved by the ethics committee of Southern Medical University, Guangzhou and all subjects or their guardians provided written consent to get blood samples for genetic testing.

### Genetic Test

**Figure 1:**
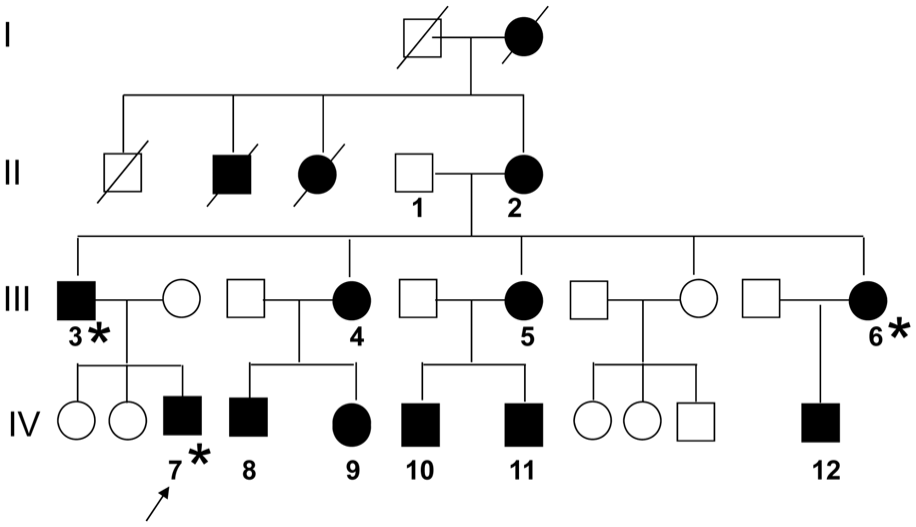
Pedigree of four generation family with EA2. Generations are shown as I to IV. Individuals affected with episodic ataxia are indicated by a black filled circle or square for females or males. Numbers (1–12) were assigned to individuals who agreed to take blood samples. The proband (III-7) is indicated by an array. DNA samples went to next generation sequencing were labeled with Asterisk (*).

Because of the clinical features overlapping among the EAs and the novel phenotype presented in this family, four known genes, CACNA1A, KCNA1, CACNB4, SLC1A3, responsible for four EAs, were sequenced by next generation sequencing that was performed by BGI (Shenzhen, China) on subject 3, 6 and 7 (Figure 1a). Experimental procedures included the following three steps. Step 1: library preparation. Genomic DNA was extracted from blood samples and fragmented. Fragments were ligated by adapters and purified. Step2: hybridization. Templates were then amplified by ligation-mediated PCR (LM-PCR) on non-captured samples, purified, and hybridized to a customized 2.1M Human Array from Roche NimbleGen (Madison, USA) for 68–72 hours. After washing, captured LM-PCR was obtained. STEP 3: Sequencing. Captured LM-PCR products were subjected to Agilent 2100 Bioanalyzer and ABI stepOne to estimate the magnitude of enrichment. After quality control, DNA libraries were bound to complementary adapter oligos grafted on the surface of the Illumina sequencing flow cell. The templates were copied from the hybridized primer by 3′ extension using a high fidelity DNA polymerase to create clonal clusters. Each captured library was then loaded on HiSeq2000 platform for sequencing. Data analysis was performed using the Illumina Bioinformatics analysis pipeline. Raw image files were processed by Illumina basecalling Software 1.7 for base-calling with default parameters and the sequences of each individual were generated as 90bp pair-end reads. The suspicious mutation was further confirmed by Sanger sequencing in all affected individuals and one unaffected member. Primers were: forward, CACNA1A-F:5′- CAGAGCCCACAAGGTCATTC- 3′, reverse, CACNA1A-R:5′-GCTCAGCCACCCTCATATTC-3′. All PCR reactions were performed as following conditions: denaturation at 94°C for 5 min; 30 cycles of 94°C for 30 s, 56°C for 30 s, and 72°C for 35 s; and a final extension at 72°C for 10 min. The predicted size of PCR products was 659 bp.

## Results

### 1. Clinic Features of a Chinese Family with EA-2

The four-generation pedigree was shown as in figure 1. A 19 year old proband (IV-7) visited our hospital in March 2009 when he experienced recurrent abdominal pain around the navel associated with limb weakness, dizziness, difficulty swallowing, dysarthria and diplopia. He had similar attacks for the past 4 years with a frequency once per 2–3 days. The symptoms lasted up to 1 h and disappeared without treatment. No precipitating factors were found. Neurological examination disclosed no obvious sign except for awkwardly rotation and drunken gait. Electroencephalogram recording revealed normal activity during attack. Brain magnetic resonance imaging was normal. During hospitalization, the patient had three attacks with 3–4 days intervals. During attacks, patients suffered abdominal pain, dizziness, dysarthria, and limb weakness. Limb tremor and upward nystagmus were observed once. The patient responded well to acetazolamide.

Family history revealed that 10 other kindred, deceased great-grandmother, deceased grandma’s sister and brother also had similar symptoms. Then, affected individuals in the family were evaluated and their symptoms and signs were described in table 1. All visited individuals were numbered (in figure 1) and their DNA samples were obtained. Except for the symptoms and signs reported in the literature, a total of five patients in this family suffered from abdominal pain during attacks (showed in table 1). Most patients recovered from attacks after rest or sleep without any treatment.

**Table 1:** Clinical features of EA-2 family.

Pt.no	Age	Age of onset	Trigger	Duration	Frequencies	Abdominalpain	Gait ataxia	Additional symptoms	interictal symptoms
2	74	5	−	several; ds	3–4/y	+	+	slurred speech, nystagmus, myokymia,nausea and vomiting	−
3	54	35	emotion, sports	6 hs	1–2/m	−	+	dysarthria, nystagmus, blurred vision, visualfield defects, seizure, myokymia, dystonia, weakness,migraine, dizziness, nausea, vomiting,sweating	
4	47	12	−	mins	1/1–4 w	+	+	dysarthria, vertigo, nausea and vomiting	+
5	44	6–7	−	up to 5 mins	1/ys	+	−	nausea and vomiting	+
6	39	7	−	till sleep	½–3 m	−	−	diplopia, headache, vertigo, lower extremitynumbness	+
7	21	15	−	1 h	1/2–3 d	+	+	dysarthria,diplopia,dizziness, nauseaand vomiting	
8	25	4	emotion, frightened sports	3–4 hs	1/1–2d	−	+	dysarthria,diplopia,defects of visualfield, myotonia,myokymia sometime,vertigo,weakness, dizziness, obvious rightarm tremor	+
9	21	17	tired	mins	1/1–2 w	−	−	diplopia, migraine, tinnitus sometime	+
10	21	7	frightened, sports	till sleep	irregular		+	dysarthria, diplopia,headaches, vertigo,myokymia, tinnituspalpitation, sweat, unable to hold items	−
11	16	5	−	hs	1/y	+	+	dysarthria, weakness, migraine, nauseaand vomiting	
12	16	13	sports	mins	1/m	−	−	dysarthria,diplopia, vertigo, tinnitus (low noise)	−

Pt no = patients number; min = minute; h = hour; d = days; w = week; m = month; y = year.

### 2. Characterization of CACNA1A Mutation

**Figure 2:**
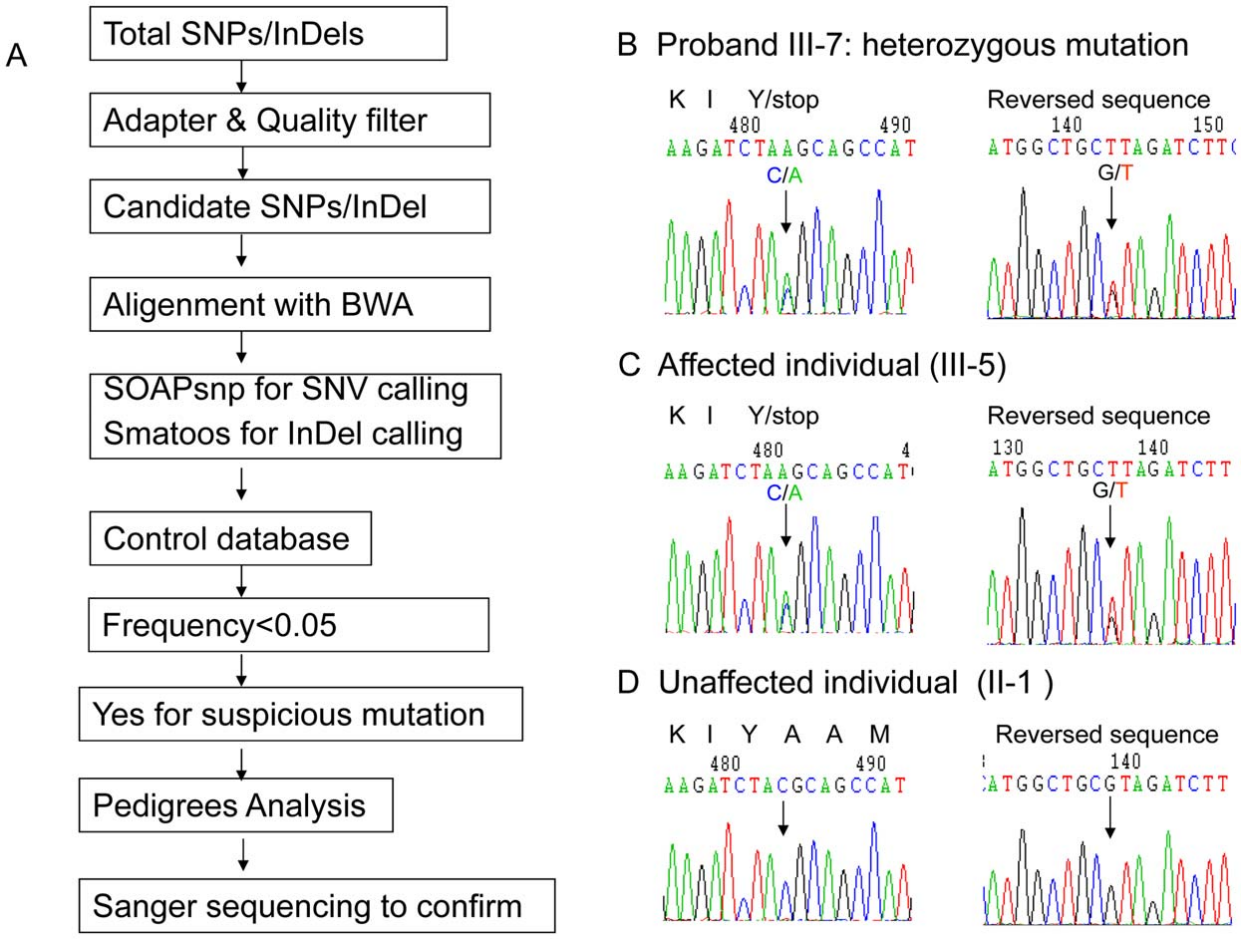
Identification of novel premature mutation (p.Tyr1957Ter) in the c-tail of CACNA1A in a Chinese EA2 family. A. Schematic of the bioinformatics analysis pipeline of data from next generation sequencing to identify nonsense mutation (p.Tyr1957Ter) in CACNA1A. SNP, single nucleotide polymorphism, InDels, insertions or deletions, BWA, Burrows-Wheeler Alignment Tool, SNV, single nucleotide variants. B. Confirmation of a heterozygous mutation in CACNA1A (c.6107C>A, NM_001127221.1, p.Tyr1957Ter, NP_001120693.1) by Sanger sequencing in the proband. C. A heterozygous C/A change at nucleotide 6107 in the affected individual D. C/C sequence at nucleotide 6107 in grandfather (I-1).

Even though the patients presented EA2 like symptoms, due to the large size of CACNA1A, lack of no hot mutation spots, and existing abdominal pain not reported in literature, we choose to sequence four known EAs responsible genes CACNA1A, KCNA1, CACNB4, and SLC1A3 with DNA samples from proband (IV-7), his father (III-3) and one aunt (III-6). We found nonsense mutation in CACNA1A (c.6107C>A, exon 40, NM_001127221.1) in all three patients. This mutation introduced a premature stop codon (p.Tyr1957Ter, NP_001120693.1 ) and caused either loss of 305 aa (amino acids) in the C-tail or nonsense-mediated mRNA decay. Sanger sequencing confirmed the heterozygous genetic defect mutation in all affected patients. The Human Gene Mutation Database Data and literature search revealed a novel mutation in CACNA1A. The sequencing raw data analysis and Sanger sequencing results were shown as in figure 2 and Tables S1.

## Discussion

Here we described a Chinese four-generation family with EA2. Recurrent attacks lasted from minutes to days and were associated with or without nystagmus, dysarthria, seizure, myokymia, dystonia, weakness, blurred vision, visual field defects, diplopia, migraine, dizziness, nausea, vomiting, sweating and abdominal pain. All symptoms or signs except for abdominal pain have been reported. Most patients experienced attacks in their childhood and teenage years except for one case, which occurred at age 35. Using next generation sequencing technology, we identified a nonsense mutation of p.Tyr1957Ter in CACNA1A. Sanger sequence confirmed all affected members harbored this heterozygous mutation.

EA2 belongs to the rare hereditary ataxia syndromes. Mutations in CACNA1A currently have been found responsible for a lot of EA2 patients. Due to the large size of the gene, mutations that widely span 47 exons, and existence of genomic rearrangement, genetic detection becomes a challenge. Low numbers of recurrent mutations were found in different studies [3], [7], [17] and detection rates from direct sequencing range from 13% to 40% [7], [18], [19]. Detections have only been carried out in advanced countries. The results show there are more than 60 mutations in EA2, the majority characterized in Caucasian families with a few identified in Japanese families [1], [7], [20]. This is the first genetic analysis study for Chinese family with EA2. However, there are lots of genetic test studies for SCA6, the allelic gene disease of CACNA1A. The detection rate for SCA6 is between 1.86–3.3%, among the largest cohort study in mainland China [21], [22].

Although truncating mutations have been reported and are the most common mutations to cause EA2, the truncated mutation of p.Tyr1957Ter in this study becomes interesting because abdominal pain has never been reported elsewhere. Five out of eleven affected members from three generations experienced with abdominal pain around the navel but only one had abnormal electroencephalogram recording. Acetazolamide, but not anti-epilepsy treatment, relieved abdominal pain of the proband. The correlation between the mutation and the phenotype remain unclear. It has recently been suggested that irregular firing of cerebellar Purkinje cells contributes to motor symptoms associated with EA2 [17].The heterozygous mutation in CACNA1A in our cases will probably produce of a mixture of wild type and truncated subunits. There are two hypotheses that suggest the genetic defection cause disease: dominant negative effect or haploinsufficiency. The former suggests that truncated proteins compete with wild type Cav2.1 and play dominant negative effect [23], [24], [25]. Some investigators support the haploinsufficiency hypothesis because the shortest truncated proteins only have domain 1 intact and large deletions are unlikely to exert a dominant negative effect [7], [10]. Since most EA2 mutations disrupt the reading frame, it is unlikely that abdominal pain is caused by “dominant negative effect” of the p.Tyr1957Ter. However, abdominal pain and episodic ataxia can be observed in many metabolic and cerebral ataxia disorders, suggesting a shared mechanism in neuroendocrine dysregulation.

In summary, a novel nonsense mutation of p.Tyr1957Ter in CACNA1A was identified in a four-generation Chinese family. Affected individuals presented typical EA2 like clinical features except for abdominal pain. Targeted sequencing by next generation sequencing technology is an ideal approach for genetic testing for large genes or syndromes with overlapping clinic features.

## Supporting Information

Tables S1 **This file includes table S1 and S2.**
(DOC)
