# Supplementary material for: Identification of a Novel Nonsense Mutation p.Tyr1957Ter of CACNA1A in a Chinese Family with Episodic Ataxia 2
Source: PLoS One. 2013 Feb 18;8(2):e56362. doi: 10.1371/journal.pone.0056362 (PMC3575407; doi:10.1371/journal.pone.0056362)
Supplement: Tables S1 — This file includes table S1 and S2. (DOC) [file pone.0056362.s001.doc]

**Supplemental tables**

**Table S1 Summary of SNPs calling for genes CACNA1A, KCNA1, CACNB4 and SLC1A3**

| **SNP Type** | **CACNA1A** | **KCNA1** | **CACNB4** | **SLC1A3** |
| --- | --- | --- | --- | --- |
| Total SNP | 61 | 5 | 13 | 6 |
| Homozygous | 25 | 1 | 4 | 1 |
| Heterozygous | 36 | 4 | 9 | 5 |
| Intron | 53 | 0 | 7 | 5 |
| Splice | 0 | 0 | 0 | 0 |
| 5-UTR | 0 | 1 | 0 | 0 |
| 3-UTR | 4 | 2 | 6 | 1 |
| CDS | 4 | 2 | 0 | 0 |
| Synonymous | 3 | 2 | 0 | 0 |
| Nonsense | 1 | 0 | 0 | 0 |
| Missense | 0 | 0 | 0 | 0 |

SNP: single nucleotide polymorphism, UTR: untranslated region

**Table S2 Summary of InDels calling for genes CACNA1A, KCNA1, CACNB4 and SLC1A3**

| **Indel Type** | **CACNA1A** | **KCNA1** | **CACNB4** | **SLC1A3** |
| --- | --- | --- | --- | --- |
| Total Iel | 11 | 0 | 3 | 3 |
| Homozygous | 0 | 0 | 0 | 0 |
| Heterozygous | 11 | 0 | 3 | 3 |
| Insertion | 3 | 0 | 0 | 1 |
| Deletion | 8 | 0 | 3 | 2 |
| Intron | 10 | 0 | 0 | 3 |
| Splice | 0 | 0 | 0 | 0 |
| 5-UTR | 0 | 0 | 0 | 0 |
| 3-UTR | 1 | 0 | 3 | 0 |
| CDS | 0 | 0 | 0 | 0 |

InDel or Indel or Iel: insertion or deletion
